# Synthesis and Characterization of Mesoporous Silica Nanoparticles Loaded with P-Cymene against Rice Bacterial Blight

**DOI:** 10.3390/nano14030250

**Published:** 2024-01-23

**Authors:** Chaonan Li, Yalan Mo, Luying Jiao, Yiping Liu, Xiaogang Li

**Affiliations:** 1 College of Plant Protection, Hunan Agricultural University, Changsha 410128, China; lcn952518@stu.hunau.edu.cn (C.L.); myl12@stu.hunau.edu.cn (Y.M.); jly150@stu.hunau.edu.cn (L.J.); ypliu619@163.com (Y.L.); 2 Hunnan Cotton Science Institute, Changde 415000, China

**Keywords:** mesoporous silica nanoparticles, p-cymene, sustained release, acute toxicity, crop safety

## Abstract

Mesoporous silica nanoparticles (MSNs) can be used as carrier materials for the controlled release of pesticides while reducing their negative environmental impact. In this study, we screened an active ingredient, p-cymene (PC), with an excellent inhibitory effect on rice bacterial blight. Subsequently, the PC was successfully loaded onto MSNs via physisorption (PC@MSNs). PC@MSNs, characterized by a regular spherical shape, smooth surface, and an MSN average size of 262.9 nm, achieved an 8.6% drug loading capacity. The release kinetics of the PC from the PC@MSNs demonstrated a sustained release (288 h) pattern influenced by drug diffusion. The efficacy of the PC@MSNs against *Xanthomonas oryzae* pv. *Oryzae* paralleled those of PC. Acute toxicity assays revealed that the PC@MSNs were less toxic to aquatic life (LC_50_ = 257.867 mg/L) and that the formulation showed no adverse effects on rice seedling growth. In summary, these results suggest that PC@MSNs can broaden PC’s scope of application in managing rice diseases.

## 1. Introduction

Pesticides are essential in modern agriculture and greatly contribute to ensuring a steady supply of food [1,2,3]. However, a significant portion of the pesticides applied in the field are eventually wasted because of low utilization rates, instability, poor dispersibility, and environmental factors such as wind, rain, and photolysis, and only a limited amount of active ingredients reach the intended targets [4,5,6,7]. Furthermore, the excessive and unscientific use of pesticides severely threatens ecosystems and human health. Traditional pesticide formulations have drawbacks, including the presence of poisonous solvents, the coarseness of particles, and inadequate dispersion. These issues contribute to reduced biological activity, lower utilization, and a shorter period of effectiveness, leading to serious contamination and damage to non-target organisms [8,9,10]. The development of innovative approaches to mitigate pesticide losses and enhance the efficacy of pesticide utilization has been necessitated by relevant factors, such as resistance, eutrophication of water bodies, short-term plant protection, and bioaccumulation [11,12].

Controlled-release formulations (CRFs) are currently a trending topic of research in the realm of pesticide formulation [13,14,15,16]. CRFs have numerous advantages such as protecting pesticides from external factors, shielding irritating odors, reducing toxicity and volatility to non-target organisms, regulating the release of pesticides, and improving adhesion and transportation capabilities of the pesticide within the target [17,18,19,20]. Over the past few years, nanopesticide formulations have emerged as a research focus in this field, providing the capability to improve utilization, mitigate toxicity to non-target organisms, and achieve a controlled release of pesticides [21,22,23,24,25]. Mesoporous silica nanoparticles (MSNs) have several advantages such as high specific surface area, tunable pore size, consistent particle size, easy surface modification, and excellent biocompatibility [26,27,28,29]. Due to these advantages, they are widely used as carrier materials for pesticides. Incorporating pesticides into MSNs not only extends their period of effectiveness but also reduces toxicity and environmental impacts on non-target organisms [30,31].

Rice is an important food crop cultivated worldwide, feeding more than half the global population [32]. Bacterial blight in rice, which is caused by *Xanthomonas oryzae* pv. *Oryzae* (*X. oryzae*), is a bacterial disease that seriously affects the yield and quality of rice [33]. Currently, copper fungicides are the most commonly used pesticides for controlling bacterial blight. The extensive use of copper fungicides not only leads to pathogen resistance but also poses a significant threat to ecosystems and human health. Therefore, the pesticide, p-cymene (PC), was filtered to control bacterial blight; this yielded satisfactory results.

**Figure 1:**
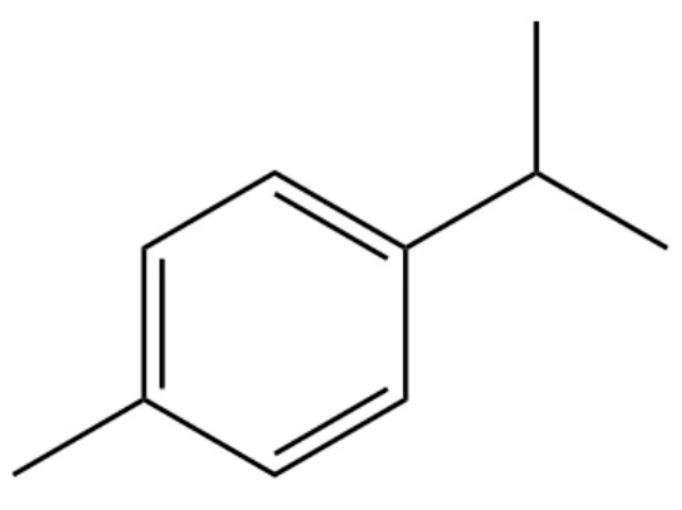
Structural formula of p-cymene.

PC is an aromatic terpene that exhibits a variety of biological activities, including anti-inflammatory, anxiolytic, antinociceptive, antioxidant, anticancer, and antimicrobial effects, and is found in over 100 plant species (Figure 1) [34,35,36,37]. PC is mainly used in medicine and rarely for crop disease control. The result of bactericidal activity (EC_50_ = 3.178 mg/L) showed that PC exhibited excellent bactericidal activity against *X. oryzae*. However, PC undergoes oxidation upon prolonged exposure to oxygen. Further, the field environment is complex, and the efficacy of PC applied in the field cannot be guaranteed. Additionally, PC can contaminate soil and groundwater, and increase the risk of food safety-related hazards when present at high levels in food products [36]. Consequently, the widespread adoption and utilization of PC in the agricultural sector is limited. Transforming PC into CRFs can diminish the required dosage and application frequency of PC, enhance its stability and safety, and address the deficiencies associated with traditional formulations.

**Scheme 1:**
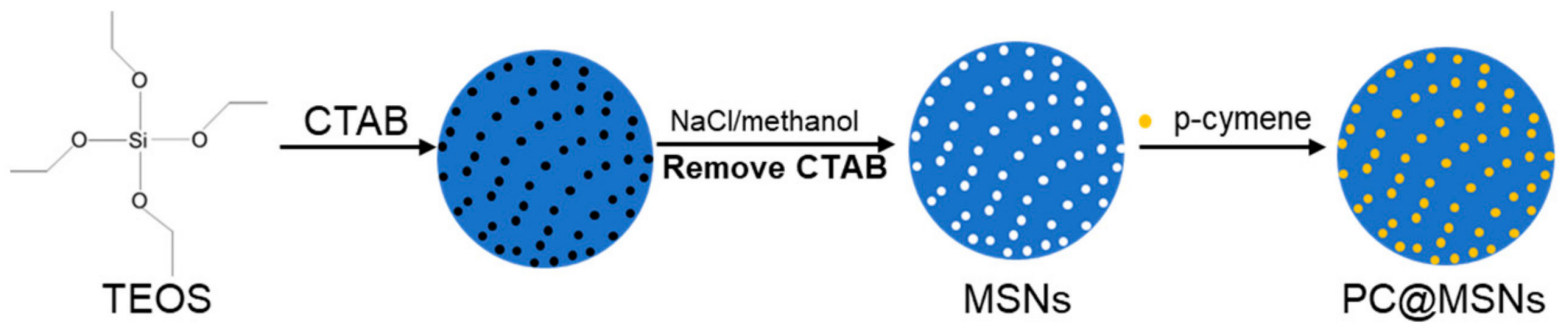
Synthetic schematic illustration of PC@MSNs.

Hence, in this research, we prepared PC-loaded mesoporous silica nanoparticles (PC@MSNs) using an impregnation method designed for the sustained release of the active component (Scheme 1). The physicochemical characteristics of the PC@MSNs were characterized. Additionally, the loading capacity, in vitro release behavior, antibacterial activity, acute toxicity to zebrafish, and biosafety in rice growth were also evaluated.

## 2. Materials and Methods

### 2.1. Materials

*X. oryzae* (PXO99) was sourced from the Laboratory of Plant Disease Control and Utilization at Hunan Agricultural University, Hunan Province, China. Hexadecyl trimethylammonium bromide (CTAB), tetraethyl orthosilicate (TEOS), and PC were procured from Macklin Biochemical Co., Ltd. (Shanghai, China). N, N-Dimethylformamide (DMF), acetic acid, sodium hydroxide (NaOH), sodium chloride (NaCl), methanol, and ethyl alcohol were procured from Sinopharm Group Chemical Reagent Co., Ltd. (Shanghai, China).

### 2.2. Synthesis of MSNs

The synthesis of MSNs was carried out following a method that has been previously documented, albeit with a few alterations implemented in this study [38]. First, CTAB (1 g) and NaOH aqueous solution (2 mol/L, 3.5 mL) were added to distilled water (480 mL) contained in a 500 mL conical flask. Subsequently, TEOS (5 mL) was slowly added to the mixture with vigorous stirring. The mixture was then stirred at 90 °C for a duration of 4 h. After being stirred, the precipitate was collected through filtration, washed with methyl alcohol and distilled water, and dried under vacuum. Second, the precipitate (0.1 g) was added to a flask containing NaCl methanol solution (50 mL), and the mixture was stirred at 70 °C for 3 h, a process that was repeated thrice. The precipitate was collected after filtration, washed with methyl alcohol and pure water, and dried under vacuum.

### 2.3. Synthesis of PC@MSNs

The PC@MSNs were prepared by impregnation. First, PC (1 g) was dissolved in methyl alcohol (50 mL) in a 100 mL conical flask. Following this, the MSNs (1 g) were added to the PC-containing solution, and stirred for a period of 24 h. The resultant PC@MSNs were washed with methyl alcohol and dried under a vacuum.

### 2.4. Characterization

The functional groups present in the specimens were examined using Fourier transform infrared spectroscopy (FTIR, Nicolet-IS 5, Thermo, Waltham, MA, USA). The analysis was conducted using the KBr disc method, with a scanning range from 4000 to 400 cm^−1^. The measurements for both the average particle size and the zeta potential were conducted using dynamic light scattering (DLS; Zetasizer Nano ZS90, Malvern, UK). The analysis of the samples’ surface composition was conducted using X-ray photoelectron spectroscopy (XPS, ESCALAB 250XI, Thermo, Waltham, MA, USA). Additionally, the N_2_ adsorption–desorption isotherms were employed to measure the surface areas, pore volumes, and pore size distributions of the specimens.

### 2.5. Microscopic Morphology Observation

The morphological characteristics of MSNs and PC@MSNs were determined using scanning electron microscopy (SEM) and transmission electron microscopy (TEM). SEM images were captured using a Sigma 300 instrument (ZEISS, Oberkochen, Germany) at an acceleration voltage of 3 kV. The TEM images were produced using a Tecnai F20 microscope (FEI, Hillsboro, OR, USA) at an acceleration voltage of 200 kV.

### 2.6. Drug Loading Content of PC@MSNs

The thermogravimetric losses of MSNs and PC@MSNs were determined using a thermogravimetric analyzer (TGA, Mettler Toledo, Zurich, Switzerland). The procedure was conducted under a nitrogen atmosphere, with the temperature within the analyzer being progressively increased from 30 to 800 °C at a rate of 10 °C per minute. The drug loading content of PC@MSNs was then calculated.

### 2.7. In Vitro Release of PC@MSNs

To evaluate the release behavior of PC from the PC@MSNs, controlled-release experiments were conducted. In the standard procedure, PC@MSNs were placed inside a dialysis bag (with a molecular weight cut-off of 3500 Da) and submerged in 200 mL of the release medium, which was a mixture of methanol and water in a 7:3 volume ratio. At predetermined time intervals, 1 mL samples of the solution were extracted and subsequently subjected to analysis using high performance liquid chromatography. Following each sampling, an equivalent volume of fresh release medium was added to maintain a consistent volume throughout the predetermined time intervals.

The quantification of drug release was performed using a high-performance liquid chromatography (HPLC) system (Waters e2695, Waters, Milford, MA, USA), outfitted with an Agilent 5 HC-C18(2) column (250 mm × 4.6 mm). The system was operated at a flow rate of 1 mL/min, with a mobile phase composed of water and acetonitrile in a 10:90 volume ratio. The injection volume was set to 10 μL, and the temperature of the column was consistently held at 30 °C. The cumulative release rate was then calculated using Formula (1).

(1) $$M=\left(Q_{t}+\frac{V_{m}}{V}\sum_{1}^{t-1}Q_{t}\right)V$$

In this context, ‘M’ represents the cumulative release amount of PC at the release time ‘t’. ‘Q_t_’ denotes the concentration of PC in the release medium during a time period from ‘t’ to ‘t − 1′. ‘V_m_’ is the volume of the release medium taken during the unit time period, and ‘V’ signifies the total release volume.

### 2.8. In Vitro Bactericidal Activity

The bactericidal activity of the PC and PC@MSNs against *X. oryzae* was assessed using the turbidity method. Specifically, bacterial fluid of *X. oryzae* (50 μL) was inoculated with nutrient broth fluid medium (50 mL) containing different concentrations (1, 2, 3, 4, 5, and 6 mg/L) of PC and PC@MSNs, respectively. The test tubes were then incubated on rotary shakers at 28 ± 1 °C for 48 h. After incubation, the absorbance of the medium was measured using an ultraviolet spectrophotometer. The fluid medium with only PC and PC@MSNs served as blank controls, and all experiments were conducted in triplicate. The concentration for 50% of the maximal effect (EC_50_) was calculated using SPSS software 27.0.

### 2.9. Acute Toxicity of PC and PC@MSNs to Zebrafish

In this study, the acute toxicity of PC and PC@MSNs to zebrafish was assessed using a semi-static toxicity test. Zebrafish were acquired from an online marketplace and maintained under laboratory conditions. In this study, zebrafish with an average length of 2.0 ± 0.1 cm were used. Prior to the commencement of the experiment, the zebrafish were allowed to acclimate to the laboratory conditions for a period exceeding seven days. Additionally, feeding was discontinued 24 h before the start of the experiment and remained so for its duration. The series of concentrations was established based on a preliminary experiment with PC. Seven zebrafish were randomly placed into tanks containing aerated water (2 L) with varying concentrations of PC and PC@MSNs. Each treatment was conducted in triplicates. The control groups containing only water and carriers were established under identical conditions. Symptoms of intoxication and mortality were monitored and recorded 24, 48, 72, and 96 h after the initiation of the test, and the deceased fish were promptly removed. The criterion for determining mortality was the absence of a response when the tail of the fish was prodded with a glass rod. Throughout the exposure period of 96 h, LC_50_ values and their 95% confidence intervals were calculated using SPSS software 27.0.

### 2.10. Safety Evaluation of Rice

Initially, the impact of PC@MSNs on rice seed germination was assessed. In the germination test, nine seeds were sterilized and positioned on a filter paper inside a Petri dish (9 cm in diameter). Water at concentrations of 50, 100, and 500 mg/L, amounting to five milliliters, was added to the Petri dish to dampen the filter paper (three replicates for each concentration). A control group was also established, which consisted of pure water. All the rice seeds were incubated in an artificial climate chamber at a temperature of 28 °C, a relative humidity of 95%, and a light/dark cycle of 12/12 h for a duration of 7 days. Each treatment was performed in triplicate. The count of germinated seeds was recorded daily, and the germination rates and potential were computed using the total number of rice seeds [39].

A moderate quantity of rice seeds was placed on a filter paper in a Petri dish for germination. Following germination, the seeds were transferred to pots containing 400 g of cultivated substrate soil, with an appropriate volume of water added to maintain a humidity level of approximately 80%. After seven days, PC@MSNs containing 100 mg of PC were applied to the soil in each pot, and pure water served as a blank control. After 21 d of treatment, the fresh weight (mg), stem length (cm), and root length (cm) were measured to assess the effects of PC@MSNs on rice growth.

## 3. Results

### 3.1. Morphological Analysis

**Figure 2:**
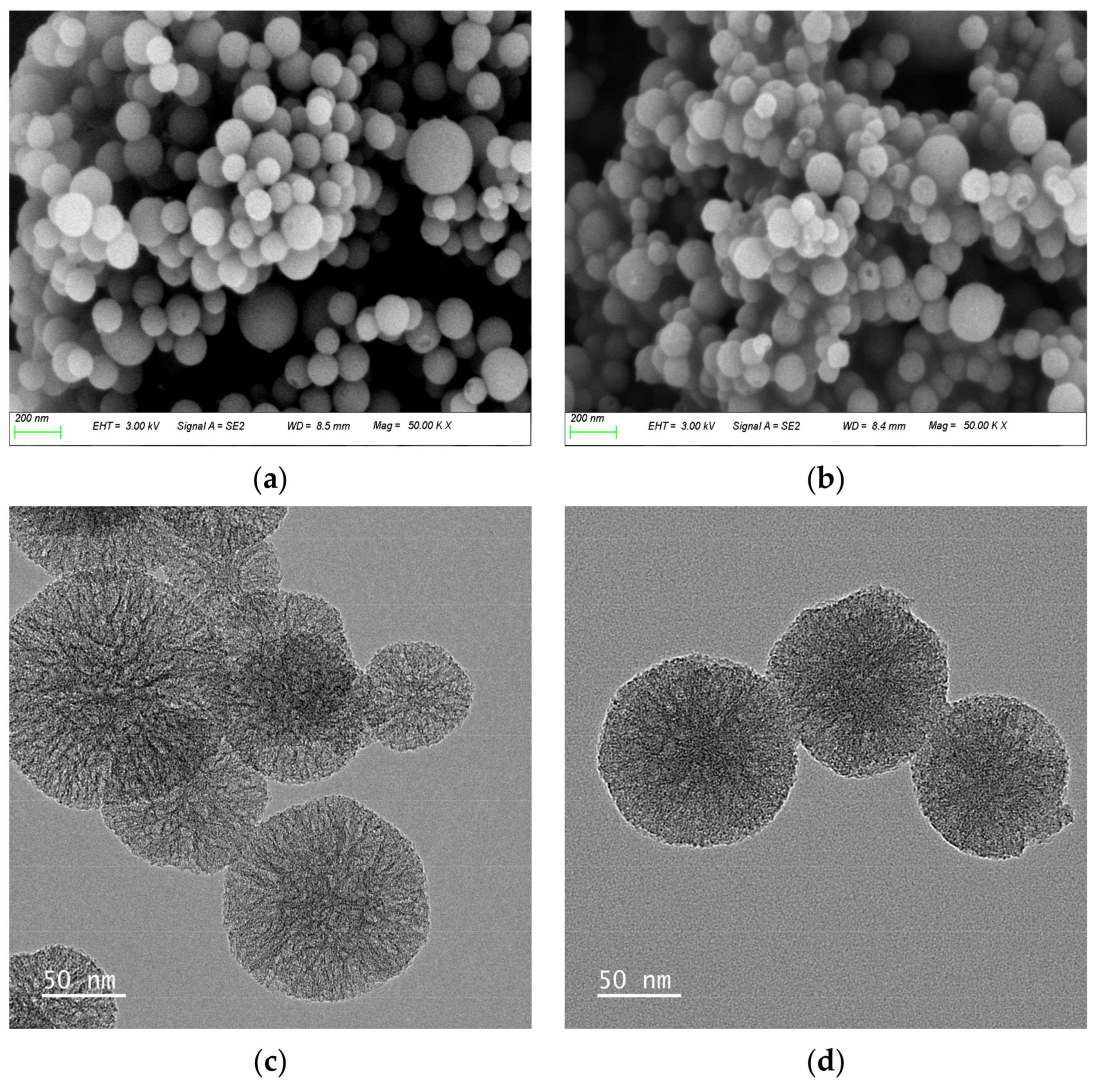
Scanning electron microscopy (SEM) images of (**a**) MSNs and (**b**) PC@MSNs; transmission electron microscopy (TEM) images of (**c**) MSNs and (**d**) PC@MSNs.

The morphology and mesoporous structure of MSNs and PC@MSNs were scrutinized using SEM and TEM. As depicted in Figure 2a,b, the MSNs exhibited a globular structure, with an average dimension of roughly 150 nm. The PC@MSNs also demonstrated a globular structure, but with an enlarged size of 260 nm. Furthermore, the internal morphology of MSNs displayed dispersed star-shaped ordered wormhole structures. However, these structures were not visible in PC@MSNs, suggesting that PC was incorporated into the mesoporous structures of the MSNs (Figure 2c,d).

### 3.2. FTIR Analysis

**Figure 3:**
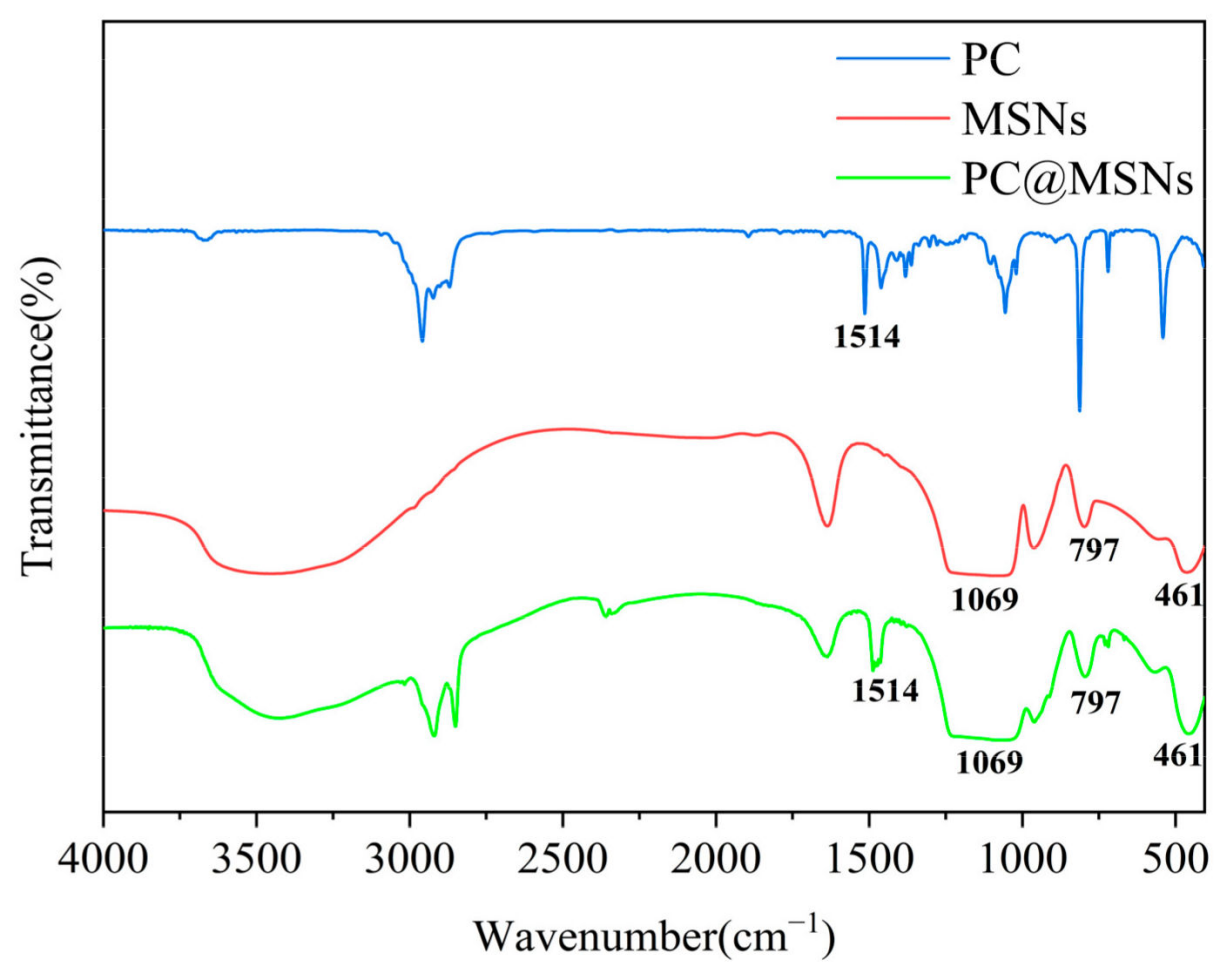
Comparison of the Fourier transform infrared (FTIR) spectra for the PC, MSNs, and PC@MSNs.

Figure 3 presents the FTIR spectra of PC, MSNs, and PC@MSNs. The PC spectrum exhibited strong absorption bands at 1514 cm^−1^, attributed to the vibration of carbonyl groups and benzene ring skeletons in PC [40]. In the case of MSNs, the peaks observed at 1069, 797, and 461 cm^−1^ were ascribed to the antisymmetric stretching, symmetric stretching, and bending vibrations in Si–O–Si, respectively [41]. The FTIR spectrum of PC@MSNs displayed a stretching vibration at 1069, 797, and 461 cm^−1^, caused by Si–O–Si, and the strong band absorption of the benzene ring at 1514 cm^−1^, also observed in PC. The attenuation of the characteristic peaks of benzene ring skeleton vibrations at 1514 cm^−1^ further substantiated the conclusion that PC was successfully coated with MSNs.

### 3.3. DLS and XPS Analysis

**Figure 4:**
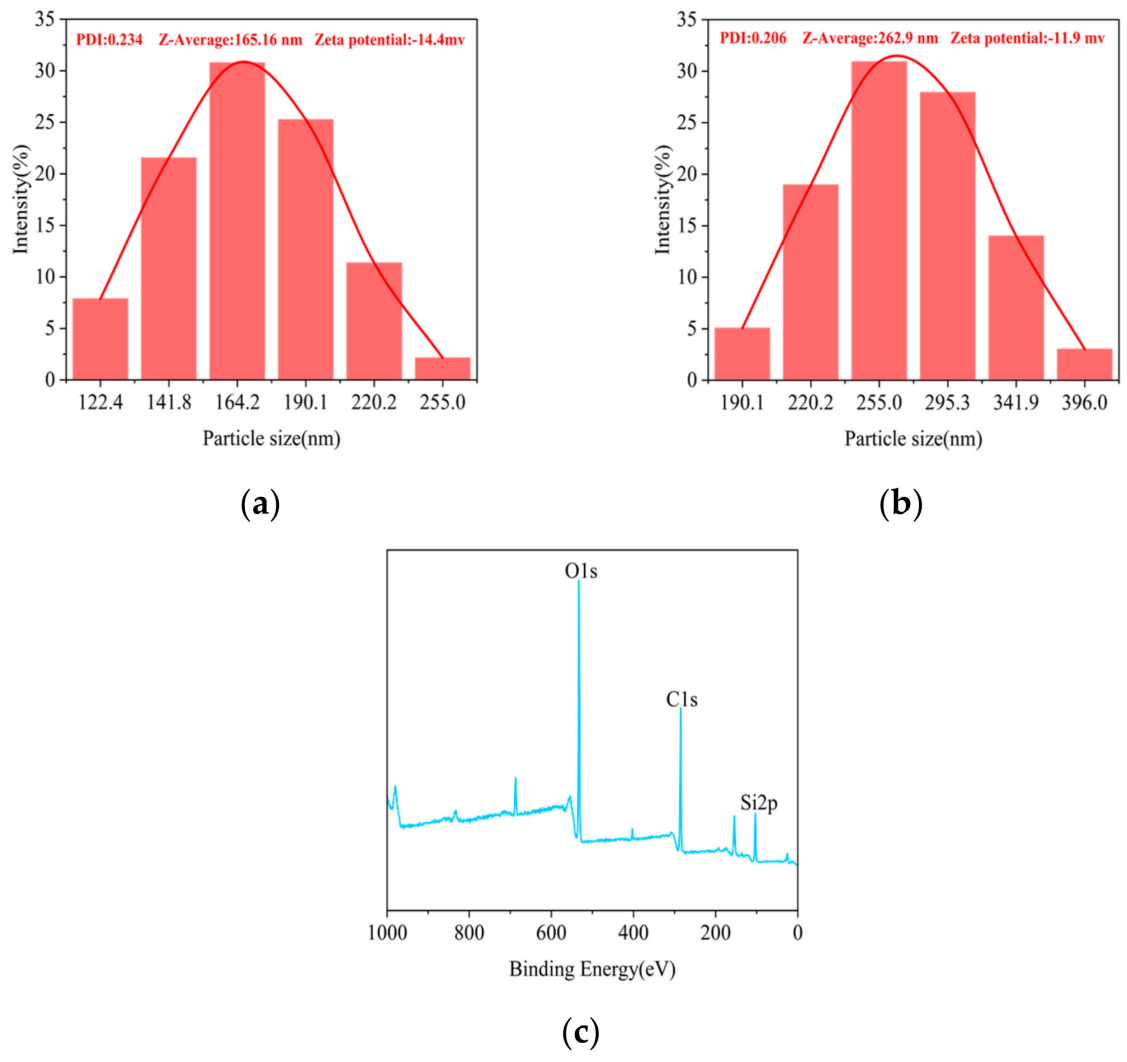
Particle size and zeta potential of (**a**) MSNs and (**b**) PC@MSNs; (**c**) X-ray photoelectron spectroscopy (XPS) spectra of PC@MSNs.

The particle size distribution and zeta potential of the MSNs and PC@MSNs are shown in Figure 4a,b. The average particle size and zeta potential of the MSNs were 165.16 nm and −14.4 mv, respectively, while the average particle size and zeta potential of PC@MSNs were 262.9 nm and −11.9 mv, respectively. In addition, the constituent elements of the PC@MSNs were determined using XPS. As indicated in Figure 4c, the presence of C, O, and Si in the PC@MSNs confirmed the successful loading of PC into the MSNs.

### 3.4. Specific Surface Area Analysis

**Figure 5:**
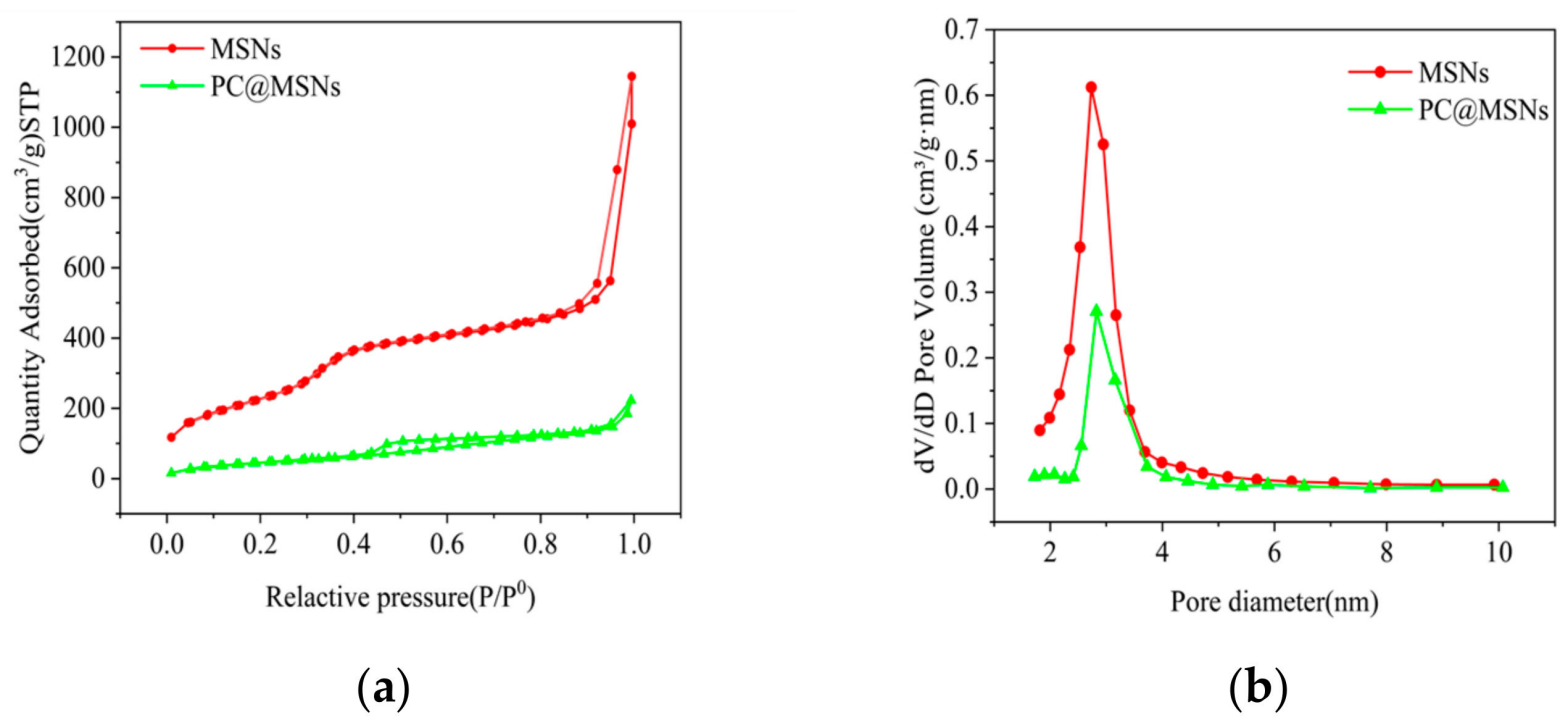
(**a**) Nitrogen adsorption–desorption isotherms of MSNs and PC@MSNs; (**b**) pore size distribution of MSNs and PC@MSNs.

Figure 5 displays the N_2_ adsorption–desorption curves and pore size distributions. The N_2_ adsorption isotherm is identified as a type IV isotherm, suggesting the presence of mesoporous structures in both MSNs and PC@MSNs (Figure 5a). Table 1 illustrates the specific surface areas, pore volumes, and pore sizes of MSNs and PC@MSNs. The MSNs had a specific surface area, pore volume, and pore size of 926.0 m^2^/g, 1.7 cm^3^/g, and 8.4 nm, respectively. Following the loading of PC into MSNs, the N_2_ adsorption capacity of the PC@MSNs was notably diminished. The specific surface area, pore volume, and pore size were reduced to 176.6 m^2^/g, 0.3 cm^3^/g, and 7.8 nm, respectively. Figure 5b reveals a significant decrease in the pore size distribution curve after drug loading, particularly at 2–3 nm. These findings confirm that PC was successfully loaded into the pores of MSNs.

### 3.5. Drug Loading Content of PC@MSNs

**Figure 6:**
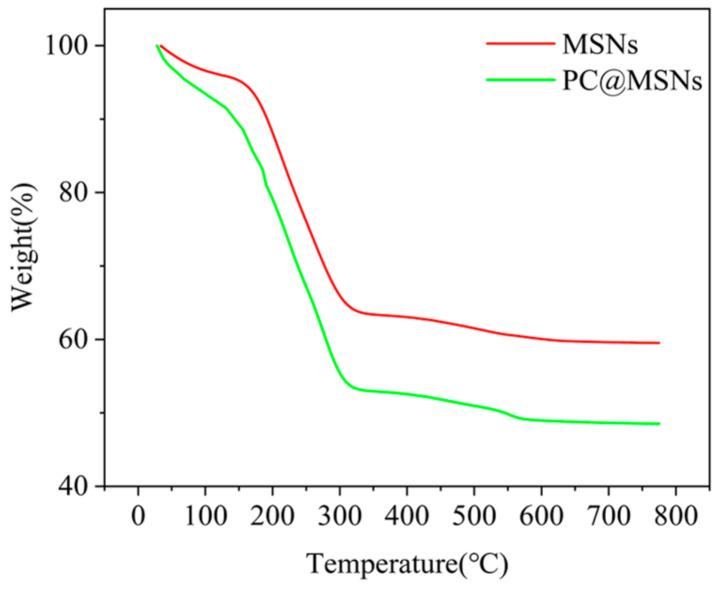
Thermogravimetric analyzer (TGA) curves for MSNs and PC@MSNs.

The TGA curves of the MSNs and PC@MSNs were obtained, and the results are presented in Figure 6. Upon reaching a temperature of 150 °C, the weight loss for MSNs and PC@MSNs was approximately 4.3% and 6.1%, respectively, which can be attributed to the removal of water from the samples. As the temperature escalated from 150 °C to 800 °C, the weight loss for MSNs and PC@MSNs increased to 38.9% and 47.5%, respectively. This weight loss resulted from the decomposition of the organic groups present in the samples [42,43,44]. The drug loading content of PC@MSNs was approximately 8.6%.

### 3.6. Release Behavior of PC@MSNs

**Figure 7:**
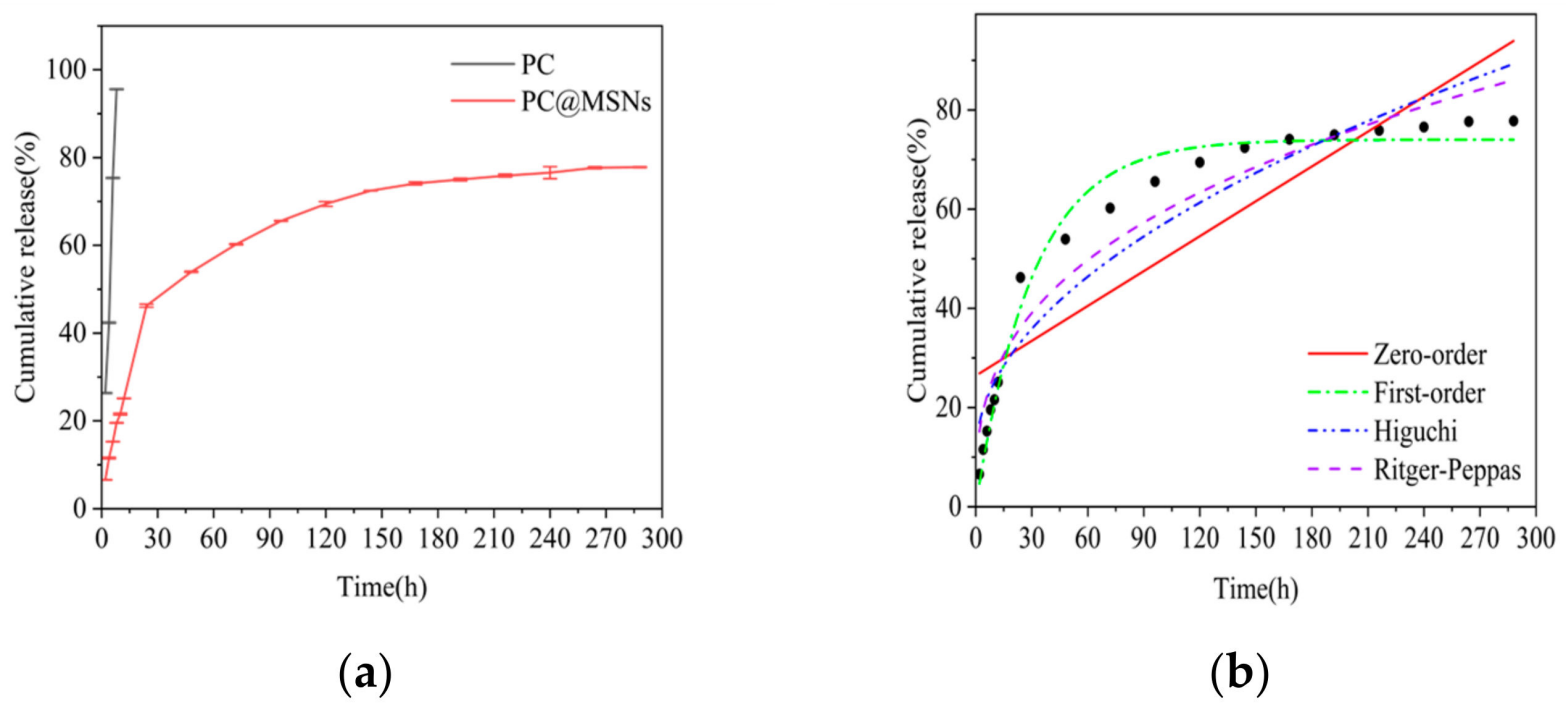
(**a**) Release performance of PC and PC@MSNs; (**b**) fit of release curve with zero-order, first-order, Higuchi, and Ritger-Peppas models.

The release behavior of PC@MSNs was investigated using the dialysis bag method. The release profiles of the PC and PC@MSNs are graphically presented in Figure 7a. The release rate of PC saw a significant increase, attaining 95.6% after 8 h. This suggests that the active component was precipitated in the medium due to a concentration difference and was fully released within 24 h. Conversely, the release of PC from PC@MSNs was more gradual, with cumulative release rates of 46.3% and 53.9% at 24 and 48 h, respectively. Following this, the release rate declined, and the sustained release time surpassed 288 h. This may be because the drug adsorbed on the surface and at the outside diameter of the pores was released first, and then, the drug was released slowly in the innermost layer.

To elucidate the release kinetics mechanism of PC@MSNs, four kinetic models were employed to fit the release curves (Figure 7b). The release curves exhibited a strong correlation with the first-order kinetic model, yielding an R^2^ value of 0.9824 for the microspheres (Table 2). These results suggest that the release of PC@MSNs adheres to the fundamental model of a sustained-release formulation. The Ritger–Peppas model, which is typically applied to erodible drug delivery systems, was used to describe the release curve. The resulting n value was less than 0.45, indicating that drug release belonged to Fickian diffusion.

### 3.7. Bactericidal Activity of PC@MSNs

To develop a more effective bactericide for the treatment of *X. oryzae*, the bactericidal activities of PC and PC@MSNs were investigated. A Thiessen copper@suspension concentrate (TC@SC) was used as the control group. As depicted in Table 3, the EC_50_ value of TC@SC was determined to be 451.482 mg/L. The EC_50_ value of PC was 3.178 mg/L, which is far below that of TC@SC. The bactericidal activity of the PC@MSNs (EC_50_ = 4.223 mg/L) was marginally lower than that of PC. This reduction was likely due to the time required for the PC@MSNs to release PC from the MSNs. These results indicate that PC and PC@MSNs had an excellent inhibitory effect against *X. oryzae* compared to TC@SC.

### 3.8. Acute Toxicity to Zebrafish

PC is widely used in medicine but rarely used in agriculture. The application of PC to control rice bacterial blight requires an evaluation of its toxicity to aquatic organisms. Therefore, we assessed the toxicity of PC and PC@MSNs to zebrafish (Table 4). Throughout the experimental period, no abnormal mortality was observed in the control or carrier-only group. The LC_50_ (96 h) values for the PC treatment and PC@MSNs were 15.165 and 257.867 mg/L, respectively. The toxicity grades of PC and PC@MSNs to zebrafish were defined as slightly toxic (≥10 mg/L). An increase in the LC_50_ values indicated that PC and PC@MSNs were less toxic to aquatic organisms. The safety of PC@MSNs for zebrafish was markedly greater than that of PC, with an approximately 17-fold increase. Compared to direct environmental exposure to PC, PC@MSNs minimized direct contact with zebrafish owing to the loading carriers, thereby further improving the safety of PC for aquatic life. In summary, the PC@MSNs have low toxicity to aquatic organisms and can promote the large-scale use of PC.

### 3.9. Plant Safety

**Figure 8:**
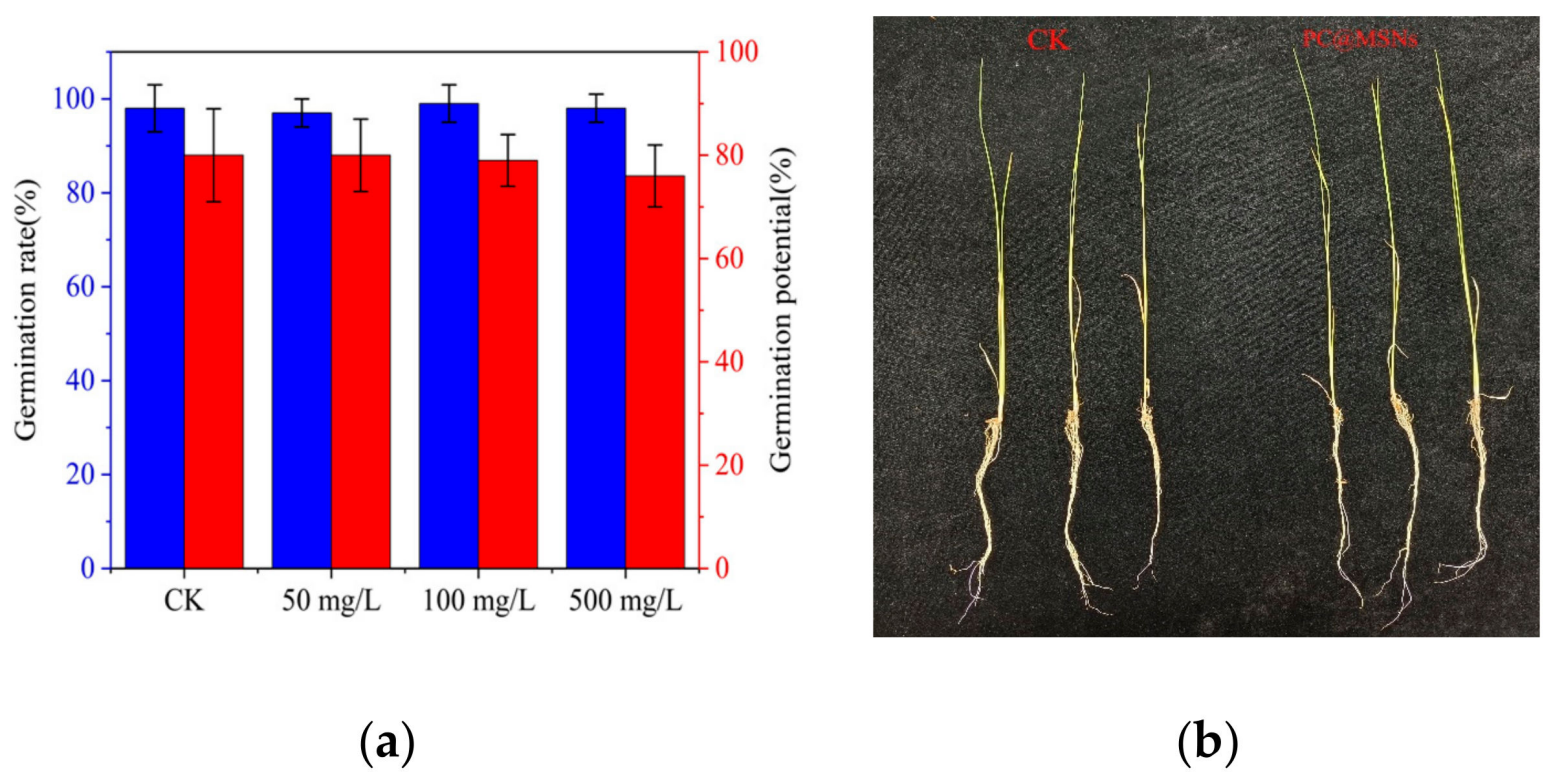
(**a**) Germination rates (in blue) and germination potentials (in red); (**b**) growth status of rice treated with PC@MSNs.

In the present study, it was observed that PC@MSNs did not have any detrimental impact on the germination of rice seeds. The germination rates for all groups, treated with varying concentrations, exceeded 95%. Furthermore, no significant disparity was noted in the germination potentials and germination rates across the different groups (as shown in Figure 8a). This suggests that the treatment with PC@MSNs does not interfere with the normal germination process of the rice seeds.

As illustrated in Figure 8b, there was no significant difference between the control group and the group treated with PC@MSNs. The results of the plant safety experiments are listed in Table 5. The fresh weight, stem length, and root length of the control rice group were 330 mg, 30.0 cm, and 17.8 cm, respectively. In the treatment group, the fresh weight, stem length, and root length of the rice were 321 mg, 29.3 cm, and 17.2 cm, respectively. After 21 d, there were no significant differences between the PC@MSN treatment group and the control group, indicating that PC@MSNs are safe for rice plants.

## 4. Discussion

Rice is one of the most important crops in the world, providing the main food source for billions of people worldwide. Bacterial blight (*X. oryzae*) is a severe bacterial disease that affects rice yield and quality. Currently, chemical agents are the most effective agents for controlling bacterial blight in rice. Among these, copper fungicide is one of the most commonly used agents to effectively control the occurrence of bacterial blight in rice. However, the excessive use of copper fungicides leads to disease resistance and poses a serious threat to the ecological environment and to human health. Many problems are associated with other pesticide formulations, including low biological activity, short duration of efficacy, and low utilization. Nanopesticide controlled-release formulations can effectively solve the problems associated with traditional pesticide formulations.

In this study, we screened a new active component (PC) to control bacterial blight in rice. The EC_50_ value of PC against *X. oryzae* was 3.178 mg/L, which was markedly lower than that of the copper fungicides (EC_50_ = 451.482 mg/L), indicating an approximately 142-fold decrease. PC is mainly used in medicine and rarely in agriculture. PC is easily volatilized and oxidized, and the field environment is complex, limiting its use in the field. Incorporating PC into nano-carrier materials not only extends its period of effectiveness but also assures its stability; hence, we prepared a nanopesticide formulation of PC, where a mesoporous silica nanoparticle served as the carrier, and loaded PC as the active pesticidal agent.

In our study, we utilized MSNs as a carrier and successfully fabricated PC@MSNs using the impregnation method. The impregnation method, a prevalent technique for drug loading, involves immersing a liquid containing active ingredients into various carriers. After a specific duration of contact, the residual liquid is separated, resulting in the attachment of the active ingredients to the solid carrier in the form of ions or compounds. For this particular research, we immersed MSNs in an ethanol solution of PC. Upon contact of the pores of the MSNs with the liquid, capillary pressure is generated due to surface tension. This pressure allows the liquid to infiltrate the mesopores, thereby securing PC within these mesopores. Additionally, intermolecular forces lead to the adsorption of PC onto the surface of MSNs.

The physicochemical characteristics of the PC@MSNs were characterized. Scanning electrons using SEM, particle size analysis, and drug-loading assessments revealed that the PC@MSNs maintained a spherical shape with a smooth surface. The average particle size of PC@MSNs was 262.9 nm, with a drug loading capacity of 8.6%. The main peaks from the FTIR scan show the similarities between the PC and the PC@MSNs, as well as the differences noted between the MSN and the PC@MSNs. The PC@MSNs had a decreased pore size compared to the MSN. The release results prove that the PC was loaded.

The bactericidal activity of the PC@MSNs was investigated, and the EC_50_ value of the PC@MSNs was determined to be 4.223 mg/L. The bactericidal activities of PC@MSNs against *X. oryzae* were similar to PC, which revealed that PC@MSNs have excellent control effects on rice bacterial blight. The result of release behavior showed that PC@MSNs had excellent slow control performance.

In addition, PC is rarely used in agriculture and its toxicity to non-target organisms is unknown. Therefore, assessing the toxicity of PC to aquatic organisms is indeed crucial if it is used for controlling bacterial blight in rice. We assessed the toxicity of PC and PC@MSNs to zebrafish. The LC_50_ (96 h) values of PC and PC@MSNs were 15.165 and 257.867 mg/L, respectively. The toxicity grades of PC and PC@MSNs to zebrafish were defined as slightly toxic (≥10 mg/L). However, high doses of PC can harm aquatic organisms. The LC_50_ of PC@MSNs was higher than that of PC, which can promote the large-scale application of PC. It can also reduce the harm caused by PC to the ecosystem and to human health.

The plant safety evaluation of PC@MSNs indicates that PC@MSNs exhibit plant safety for rice. The PC release exhibited an instant bolus dose which contributes to the observed toxicity. However, the PC@MSNs exhibited a slight initial bolus dose followed by a sustained controlled release, which explains why the pesticide effect is observed, and why it is sustained for a longer overall time period, with lower toxicity. This release from the formulation proves that PC has great potential as a pesticide once it is in the correct formulation, even though it has barely been used in agriculture.

This nanopesticide formulation is poised to broaden the scope of application of PC for managing rice diseases and enhance its commercial viability, marking a development of considerable practical significance. However, there are still many problems with the application of PC in agriculture, such as toxicity to other non-target organisms, and further evaluation is needed to confirm whether PC can really be employed for the purposes of disease control.

## 5. Conclusions

This study introduced PC@MSNs, a nanopesticide formulation that has excellent inhibitory effects on rice bacterial blight. Studies on the in vitro release mechanism indicated that PC@MSNs exhibit commendable controlled release capabilities, with PC release modulated by drug diffusion. Moreover, in vitro bactericidal activity assays demonstrated that the PC@MSNs showed excellent efficacy against bacterial blight. The PC@MSNs did not impede the normal growth of rice plants. In biosafety evaluations, the PC@MSNs exhibited low toxicity to zebrafish after 96 h. The PC@MSNs, as a sustained-release nanopesticide formulation, show significant potential for managing rice bacterial blight. The findings from this study open up new avenues for research incorporating PC into the agricultural field.

## Figures and Tables

**Table 1:** Specific surface area, pore volume, and average pore diameter of MSNs and PC@MSNs.

	MSNs	PC@MSNs
Specific Surface Area (m^2^/g)	926.0	176.6
Pore Volume (cm^3^/g)	1.7	0.3
Average Pore Diameter (nm)	8.4	7.8

**Table 2:** Data fitting models for PC@MSNs.

Fitting Method	Equation	R^2^
Zero-Order Fitting	Q = 25.45066 + 0.2344t	0.75217
First-Order Fitting	Q = 74.04867(1 − e^0.03261t^)	0.9824
Higuchi Fitting	Q = 10.40539 + 4.64955t^1/2^	0.90414
Ritger–Peppas Fitting	Q = 11.91204t^0.34917^	0.93925

**Table 3:** Regression equations representing the virulence of TC@SC, PC, and PC@MSNs towards *X. oryzae*.

Treatment	Equation	R^2^	EC_50_ (mg/L)	95% Confidence Interval
TC@SC	Y = −8.96 + 3.38x	0.983	451.482	415.59–497.913
PC	Y = −1.01 + 2.01x	0.996	3.178	2.809–3.599
PC@MSNs	Y = 1.89 + 3.01x	0.989	4.223	3.863–4.667

**Table 4:** LC_50_ values for PC and PC@MSNs after treatment of zebrafish.

Treatment Time (h)	LC_50_ (95% Confidence Interval) (mg/L)	
	PC	PC@MSNs
24	16.443(16.179–16.675)	259.511(256.984–264.874)
48	15.992(15.769–16.254)	258.412(256.367–262.952)
72	15.624(15.422–15.922)	258.023(255.066–261.876)
96	15.165(14.986–15.533)	257.867(256.986–258.453)

**Table 5:** Plant safety evaluation of PC@MSNs.

Treatment	Fresh Weight (mg)	Stem Length (cm)	Root Length (cm)
CK	330 ± 0.4	30 ± 1.0	17.8 ± 0.6
PC@MSNs	321 ± 0.3	29.3 ± 0.7	17.2 ± 0.5

## Data Availability

Data are contained within the article.
